# Establishing a protocol for single cell transcriptome sequencing of the rat brain

**DOI:** 10.1186/1471-2105-15-S10-P27

**Published:** 2014-09-29

**Authors:** Hao Chen, Burt M Sharp

**Affiliations:** 1 Department of Pharmacology, University of Tennessee Health Science Center, Memphis, TN 38106, USA

## Background

The rat is a widely used model for neuroscience research, particularly for complex behavioral phenotypes. We tested the feasibility of RNA-seq analysis of single cells located in the dopaminergic midbrain region, which is critical for motivated behaviors.

## Materials and methods

Brain tissue of interest from adult Sprague-Dawley rats was obtained in bilateral punches from fresh 1 mm thick coronal sections, using a 3D-printed device for guidance. The method of Guez-Barber et al. [1] was then followed to dissociate cells. Using flow cytometry, we determined that cell viability was 93-95% and neurons (i.e., NeuN+) were approximately 50%. Cell suspensions were separated in the C1 single-cell auto prep instrument into individual cells. cDNA synthesis and amplification were then performed for each cell in the integrated microfluidic circuits. Fourteen cells were selected for whole transcriptome sequencing using the Ion Proton instrument based on their morphology, positive PCR detection of marker genes, and quantity of cDNA. The Ion Torrent Suite was used to map the reads (~150bp in length) to the rat reference genome (rn5).

## Results

On average, 3,928±434 genes were detected per cell (excluding one cell that failed at the library prep step and one at the sequencing step), and a significantly greater number of genes (i.e., 5,226±54) were detected from three cells with higher cDNA content compared to the other nine cells. Only one cell expressed the stress-marker, cFos. Three cell types can be identified: neurons (expressing Th, Slc6a3, Slc17a7, Gad2, etc, n=3), astrocytes (expressing Gfap, S100a13, n=5), oligodendrocytes (expressing Olig1 and Olig2, n=3). The phenotype of one cell cannot be defined.

## Conclusions

In summary, we have established a workflow that allows the transcriptome of single neurons and glia from adult rat brain to be sequenced. Future work will focus on further improving the RNA yield.
